# Empirical Evidence for Various Evolutionary Hypotheses on Species Demonstrating Increasing Mortality with Increasing Chronological Age in the Wild

**DOI:** 10.1100/tsw.2008.36

**Published:** 2008-02-19

**Authors:** Giacinto Libertini

**Affiliations:** Independent Researcher, via Cavour 13, Caivano 80023, Naples, Italy

**Keywords:** actuarial senescence, aging, evolution, mortality rate, telomere

## Abstract

Many species show a significant increase in mortality with increasing chronological age in the wild. For this phenomenon, three possible general hypotheses are proposed, namely that: (1) it has no adaptive meaning; (2) it has an adaptive meaning; (3) the ancestry is the pivotal determinant. These hypotheses are evaluated according to their consistency with the empirical evidence. In particular, (1) the existence of many species with a constant, or almost constant, mortality rate, especially the so-called “animals with negligible senescence”; (2) the inverse correlation, observed in mammals and birds in the wild, between extrinsic mortality and the proportion of deaths due to intrinsic mortality; (3) the existence of highly sophisticated, genetically determined, and regulated mechanisms that limit and modulate cell duplication capacities and overall cell functionality. On the whole, the hypothesis of an adaptive meaning appears to be consistent with the empirical evidence, while the other two hypotheses hardly appear compatible.

## INTRODUCTION

In this paper, peculiar life tables, in which longevity is clearly programmed (e.g., aphagous insects with defective mouthparts or digestive organs in the adult state, which are thus incapable of feeding[1]) or is strictly linked to programmed events (e.g., semelparous animal and plant species[1]), are not considered.

In the more common cases, disregarding the early stages of life (development and growth of the individual), which, for various reasons, usually have a high mortality rate, not necessarily related to adult mortality, the life table of a species in the wild is roughly described by Weibull's equation[2]:

$$m_t = m_0 + \alpha \cdot t^{\beta} \quad (1)$$

in which t is the time, $m_t$ is the overall mortality rate at time t, $m_0$ is the mortality at its initial lowest value (at the time defined 0 after the early stages of life), α and β are two constants.

Many species show a significant increase in mortality with increasing chronological age in the wild[1,2,3,4,5,6,7], referred to using the acronym “IMICAW”[8] or as “actuarial senescence in the wild”[7]. An increasing mortality in artificial conditions at ages that are inexistent in the wild is in no way defined by, or considered to be, a synonym of IMICAW[8,9].

For many other species in the wild, there is a negligible fitness decline; i.e., there is a constant, or almost constant, mortality rate, with a modest increase in some species, due to the effects of injury damages accumulation ($m_w$), or, in some other rare cases, a modest decrease (negative senescence), due to the progressive increasing body mass that reduces the possibility of being predated, or to other factors[10,11,12].

Species of this second group have been defined as non-IMICAW species[8] and, in more restricted cases where the mortality rate is low and survivors reach very old ages, as “animals with negligible senescence”[1].

The IMICAW condition is illustrated in Fig. 1 while the non-IMICAW condition is shown in Fig. 2.

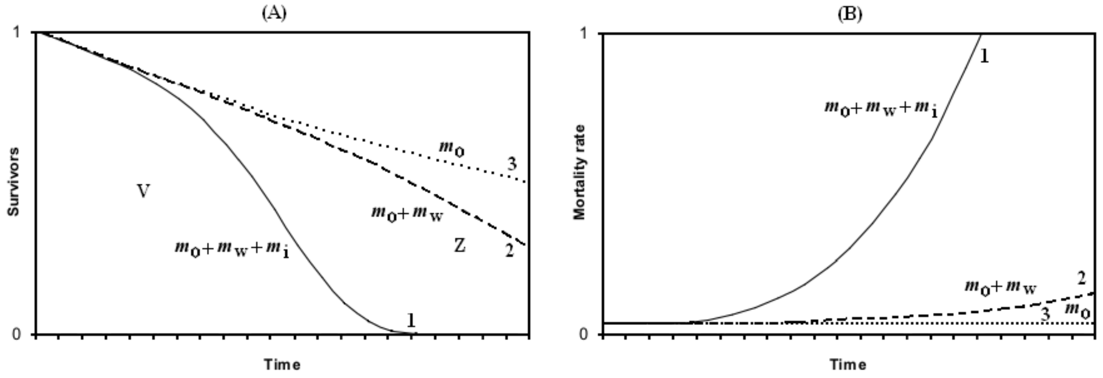

**FIGURE 1.** IMICAW condition. (A) Life table in the wild of an IMICAW species, determined by $m_0 + m_w + m_i$ (Curve 1); hypothetical life tables with the action of $m_0 + m_w$ only (Curve 2) or with the action of $m_0$ only (Curve 3); V is the area delimited by Curve 1; Z is the area between Curves 1 and 3. (B) Mortality rate in the wild of an IMICAW species, namely $m_0 + m_w + m_i$ (Curve 1); $m_0 + m_w$ (Curve 2); $m_0$ (Curve 3).

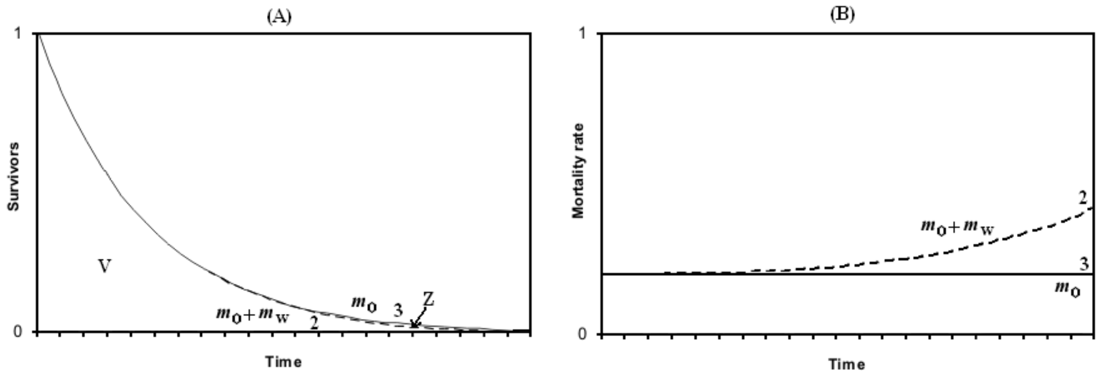

**FIGURE 2.** Non-IMICAW condition. (A) Life table in the wild of a non-IMICAW species, determined by $m_0 + m_w$ (Curve 2); hypothetical life table with the action of $m_0$ (Curve 3) only; V is the area delimited by Curve 2; Z is the area between Curves 2 and 3. (B) Mortality rate in the wild of a non-IMICAW species, namely $m_0 + m_w$ (Curve 2); $m_0$ (Curve 3).

Fig. 1B illustrates that for an IMICAW species, the overall mortality in the wild is the sum of $m_o$ (Curve 3), plus $m_w$, if present (Curve 2), plus mortality not strictly due to extrinsic factors and therefore defined as intrinsic mortality ($m_i$, Curve 1). The sum of $m_0$ and $m_w$, which are factors due largely to environmental or extrinsic causes, is defined as extrinsic mortality ($m_e$).

Fig. 1A shows the life table in the wild of an IMICAW species (Curve 1) and two hypothetical life tables: the first (Curve 3) with the action of $m_0$ only and the second (Curve 2) with the action of $m_0 + m_w$ only, that is $m_e$. The area delimited by the life table (Curve 1) is indicated with V, while the area between Curves 1 and 3, namely the decrement of the area delimited by Curve 3, causing the overall mortality increment ($m_i + m_w$), $\alpha \cdot t^{\beta}$ in Weibull's equation, is indicated with Z.

In Fig. 2 (non-IMICAW condition), Curve 1 is absent because, by definition, there is no $m_i$. The area Z in Fig. 2A, namely the difference between Curves 2 and 3, determined only by the increment of $m_w$ shown in Fig. 2B, is small.

## TOPIC

As regards the evolutionary meaning of the IMICAW phenomenon, three main general hypotheses have been proposed:

1. **Nonadaptive hypothesis** — The increase of mortality has no adaptive value and is caused by various harmful factors that limit the mean duration of life (ML):

- Harmful mutations accumulated from generation to generation[13,14,15,16,17]
   - Antagonistic pleiotropic genes[18,19]
   - Physiological, biochemical, or environmental constraints[20,21]

Their effects are partially balanced by the selection deriving from the advantages of a greater ML. This hypothesis is illustrated in Fig. 3.

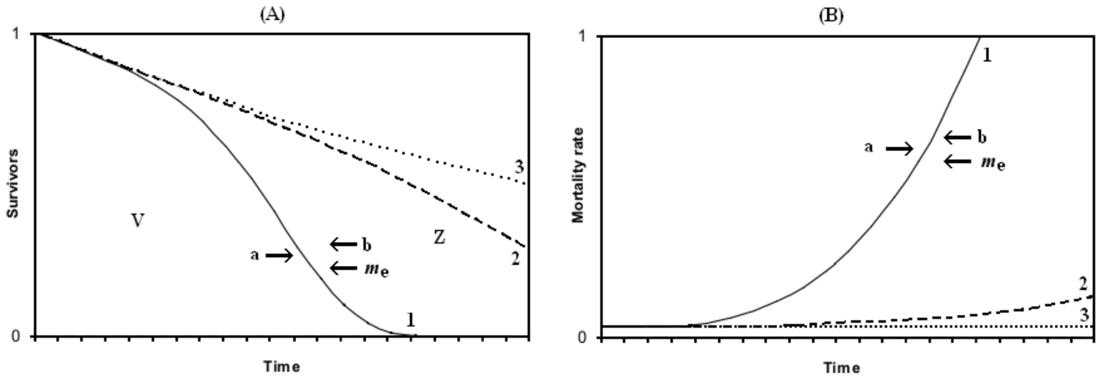

**FIGURE 3.** Nonadaptive hypothesis. The curves are the same as in Fig. 1. (A) The life table of an IMICAW species (Curve 1) is influenced by [a] = selection against ML-limiting factors; [$m_e$] = extrinsic mortality (= $m_0$+$m_w$); [b] = ML-limiting harmful factors. The hypothetical life tables without $m_i$ or without $m_i$+$m_w$ (Curve 2 and Curve 3) are influenced only by the variations of $m_0$+$m_w$ and of $m_0$, respectively. (B) Mortality rate curves are influenced by the same factors.

2. **Adaptive hypothesis** — An increase in mortality rate means a decline in fitness and reproductive potentialities, and this is always negative for individual selection. However, considering other supraindividual selective mechanisms (e.g., kin selection), it has been hypothesized that the increment in mortality could have an adaptive value[8,9,22,23,24]. Consequently, there would be a balance between selection for genes reducing the mean duration of life, namely IMICAW-causing factors, and selection against them. This hypothesis is illustrated in Fig. 4.

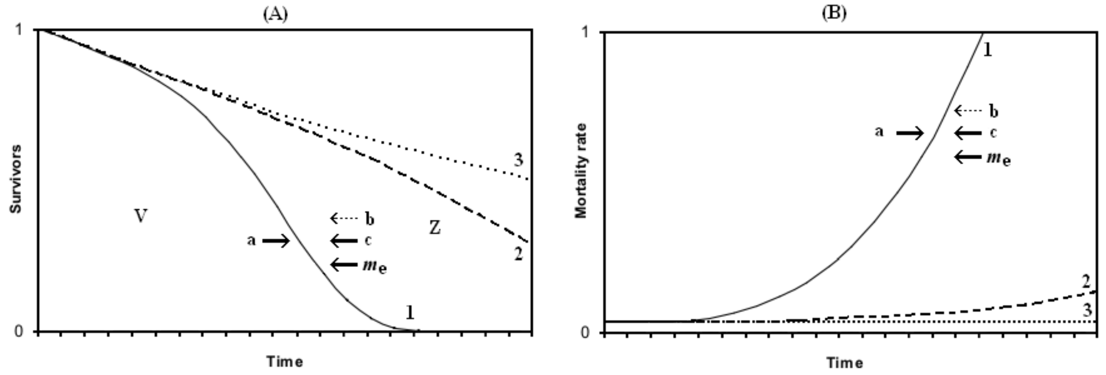

**FIGURE 4**. Adaptive hypothesis. The curves are the same as in Fig. 1. (A) The life table of an IMICAW species (Curve 1) is influenced by [a] = selection against ML-limiting factors; [c] = hypothesized advantage of the IMICAW phenomenon causing intrinsic mortality [$m_i$]; [$m_e$] extrinsic mortality (= $m_0$+$m_w$); while [b] = ML-limiting harmful factors (see nonadaptive hypothesis) is considered negligible (dashed line). (B) Mortality rates are influenced by the same factors as in Fig. 4A.

3. **Historical hypothesis** — The major determinant of life tables is the ancestry of each phylum or subphylum, or group of species, i.e., the phylogenetic history of each species, and selective factors have importance only in modulating these ancestral patterns[25].

The aim of this paper is not the exposition or discussion of theories based on the above-mentioned hypotheses, but the description of three sets of empirical data and the evaluation of their consistency with each of the three hypotheses.

## EMPIRICAL EVIDENCES

### Animals with Negligible Senescence

#### *Empirical Evidence*

If we disregard the possible modest increase in mortality due to the effects of injury damages accumulation ($m_w$), or the mortality decrease in cases of negative senescence, many species in the wild show no mortality increase; that is to say, their fitness is stable at ages found in the wild[1]. For the same species reared in artificial conditions with low mortality, starting from ages rarely or never found in the wild, it is common to find a progressive decay in physiologic functions[1] usually referred to using the same imprecisely defined[26] term “aging” with which fitness decline in IMICAW species is sometimes described[2].

The **nonadaptive hypothesis** does not predict the existence of non-IMICAW species, because IMICAW-causing harmful factors are considered as universal. We have two general cases:

1. For species with a short ML in the wild, fitness stability is explained by the simple argument that life-limiting factors do not have enough time to carry out their action. Moreover, such short-living species in captivity, in artificial conditions of low mortality, demonstrate an increment of mortality with increasing chronological age (defined by its acronym IMICAC[8]), which is taken as proof of this explanation.
2. For species whose individuals survive up to considerable ages in the wild and that, at more advanced ages found in the wild, show no detectable reduction in fitness, i.e., for “animals with negligible senescence” (e.g., rockfish, sturgeon, turtles, bivalve mollusks, certain perennial trees and, possibly, lobsters[1]), this seems genuinely unexpected and provokes considerable doubts[27]. These cases need to be explained in terms of physiologic peculiarities; that is to say, for the nonadaptive hypothesis, such animals and plants must be considered as exceptions justified by further particular hypotheses. However, particular optimization models of life-history strategies, based on the assumptions of the disposable soma theory[20,21], have been developed to justify even those cases in which survival increases at more advanced ages[12].

In short, for the nonadaptive hypothesis, IMICAW absence is, in the first case, justified by the presence of IMICAC, a different phenomenon, while in the second case, particular models or undocumented protecting factors should explain these exceptions.

The **adaptive hypothesis** for species in conditions unfavorable to IMICAW simply predicts the absence of the IMICAW phenomenon. Therefore, a theory based on the adaptive hypothesis, and which does not propose universal IMICAW-favorable conditions, predicts the existence both of IMICAW and of non-IMICAW species, and so could be consistent with the empirical data.

It must be stressed that, in the case of a non-IMICAW species displaying the IMICAC phenomenon in protected conditions, this is by no means the same thing as the IMICAW phenomenon or a retarded form thereof, and neither is it proof that a non-IMICAW species becomes an IMICAW species in protected conditions. By definition, the IMICAW phenomenon exists only in the wild and is subject to natural selection, while these statements are invalid for the IMICAC phenomenon[8,9].

As very well-known examples of short-lived, non-IMICAW species displaying the IMICAC phenomenon in protected conditions, we have *Caenorhabditis elegans*, of which the longevity "under more natural conditions is reduced up to 10 fold compared with standard laboratory culture conditions"[28] and of which few individuals in the wild remain fertile after 10 days[29], and *Drosophila melanogaster*, which, in the wild, has a reported adult lifespan of 10–12 days[1]. Another documented example is the spider *Frontinella pyramitela*, which, in the wild, lives for less than 3 weeks after sexual maturity and shows no evidence of age-related mortality acceleration. In the laboratory, this arachnid lives up to several months, with survival curves showing the IMICAC phenomenon referred to using the generic term "senescence"[1]. In all these cases, the increasing mortality observed in the laboratory is an artifact and cannot, therefore, be caused by natural selection or be used as a reliable animal model for the IMICAW phenomenon, which is subject to natural selection.

The **historical hypothesis** originates simply from the observation that some groups of phylogenetically related species display the IMICAW phenomenon, while other groups display the non-IMICAW condition, or particular forms of life table (e.g., semelparity and then rapid senescence, copulation, and death, etc.[1]). A species that does not obey the rule of its group is considered to be an explicable exception. Therefore, the historical hypothesis justifies both IMICAW and non-IMICAW species, but it is self-evident that the predicted correlation between phylogeny and IMICAW or non-IMICAW or other conditions needs a precise verification.

## Inverse Correlation between Extrinsic Mortality Rate and Proportion of Deaths due to Intrinsic Mortality

### *Premise*

Studying mortality increments in the wild, the sum $m_i + m_w$ is calculable, but the distinction between $m_i$ and $m_w$ is not directly assessable.

For the following discussion, it must be noted that the effects of the accumulation of injury damages ($m_w$) are, by definition, directly related to the severity of environmental conditions, namely $m_0$. Consequently, in the study of possible relations between extrinsic mortality ($m_e$), equal to $m_0 + m_w$, and intrinsic mortality ($m_i$) or derived parameters: if in available data $m_w$ is not with the first term, but with the second term, then this could reinforce a direct relation, should one exist, or, otherwise, simulate a direct relation. On the other hand, it could diminish, but not create, an inverse relation.

For this reason, a significant inverse relation between $m_0$ and $m_i + m_w$, if observed, will be considered as indicative of a significant inverse relation between $m_0$ and $m_i$.

However, the influence of $m_w$ cannot be great, because when $m_0$ and $m_w$ have their greatest values, the proportion of deaths due to $m_i + m_w$ is only 3%[2] and, therefore, the value of $m_w$ cannot be greater than this value.

So, in this section the effects of $m_w$ will be considered as negligible.

## Empirical Evidence

From the observational data on mammal and bird life tables in the wild, reported on and analyzed in an authoritative and documented review[2], some very interesting relations can be deduced.

The first, (Fig. 5), described by Ricklefs[2], is a significant ($p < 0.01$) positive relation between $m_0$ and the increment in mortality rate ($m_i$).

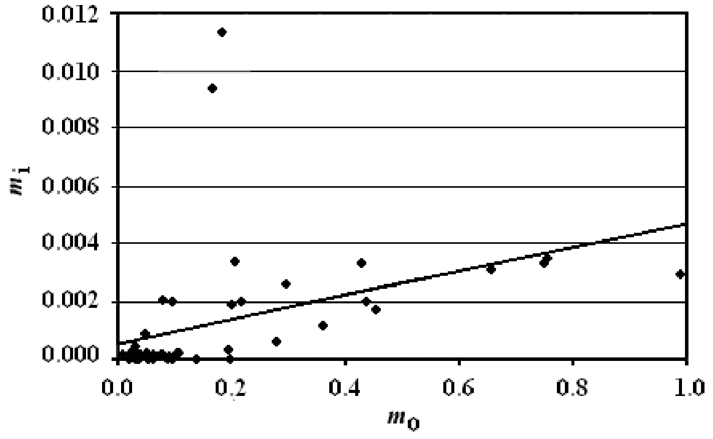

**FIGURE 5.** Direct significant correlation (linear regression, r = 0.390813535, t = 2.81634826, $p < 0.01$) between $m_0$ and $m_i$. Data are from Ricklefs[2], Appendix A, Tables A1 e A2 (pp. 36–38), and refer to 26 species of mammals and 18 of birds. One species, *Sylvilagus floridanus* (MF), has been excluded because the reported value of $m_0$ (1.225) is clearly a misprint. Correlation is significant as well for mammals and birds considered separately.

The second (Fig. 6), also described by Ricklefs[2], is a significant ($p < 0.01$) inverse relation between $m_0$ and the proportion of deaths due to $m_i$, namely the ratio Z/(Z+V).

The third (Fig. 7), not described by Ricklefs[2], is a significant ($p < 0.001$) inverse relation between $\log_{10}(m_0)$, or $m_0$, and the survivors at the age t* when the total increment in mortality ($m_i$) becomes greater than $m_0$.

The apparent contradiction between the positive relation of $m_0$ with $m_i$, and the negative relation of $m_0$ with the proportion of deaths due to $m_i$, becomes understandable after a careful examination of the third relation. In fact, comparing two very different life tables, the first with a low $m_0$ (Fig. 8A) and the second with a high $m_0$ (Fig. 8B), we can observe that in the first case, at age t* survivors are about 85%, while in the second case, $m_i$ increases more quickly, but at age t* there are no survivors. Therefore, in the first case, ML is strongly influenced by the increment in mortality rate, while in the second case, its influence is minimal.

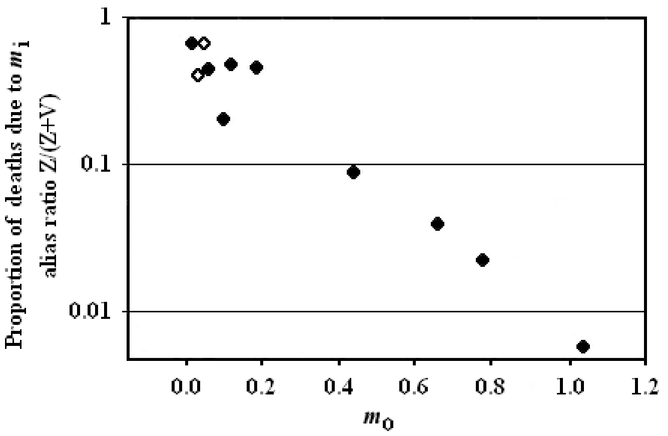

**FIGURE 6.** Inverse significant correlation (linear regression, r = –0.758708, t = –3.494043, $p < 0.01$) between $m_0$ and the proportion of deaths due to $m_i$, or ratio Z/(Z+V). Data are from Ricklefs[2], Table 2 (p. 30). Ricklefs' Fig. 7 (p. 34) has been redrawn. Ordinates are in logarithmic scale. Open symbols refer to mammal species, solid symbols to bird species.

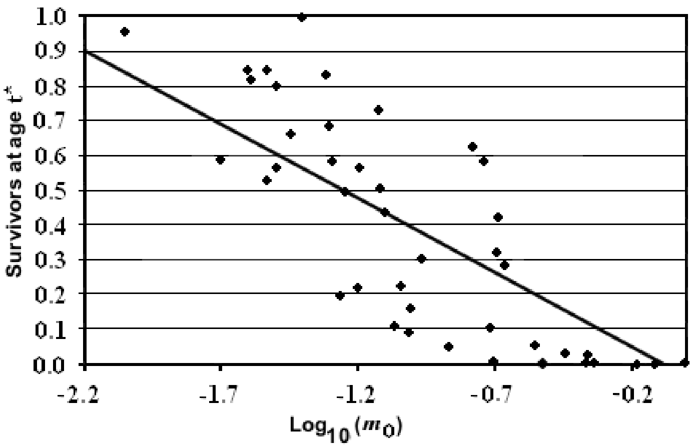

**FIGURE 7.** Inverse significant correlation (linear regression, r = –0.820448404, t = –9.519054012, $p < 0.001$) between $\log_{10}(m_0)$ and the survivors at age t* when the total increment of mortality ($m_i$) becomes greater than $m_0$. The relation has been calculated using the same data as in Fig. 5. Correlation is significant as well for mammals and birds considered separately. Substituting $\log_{10}(m_0)$ with $m_0$, statistical analysis gives r = –0.659414466, t = –5.818275394, $p < 0.001$.

For the **nonadaptive hypothesis**: "The principal determinant in the evolution of longevity is predicted to be the level of extrinsic mortality. If this level is high, life expectancy in the wild is short, the force of selection attenuates fast, deleterious gene effects accumulate at earlier ages, and there is little selection for a high level of somatic maintenance. Consequently, the organism is predicted to be short lived even when studied in a protected environment. Conversely, if the level of extrinsic mortality is low, selection is predicted to postpone deleterious gene effects and to direct greater investment in building and maintaining a durable soma"[30]. That is to say, for the nonadaptive hypothesis, with a greater $m_e$, the value and the influence of [b] should increase.

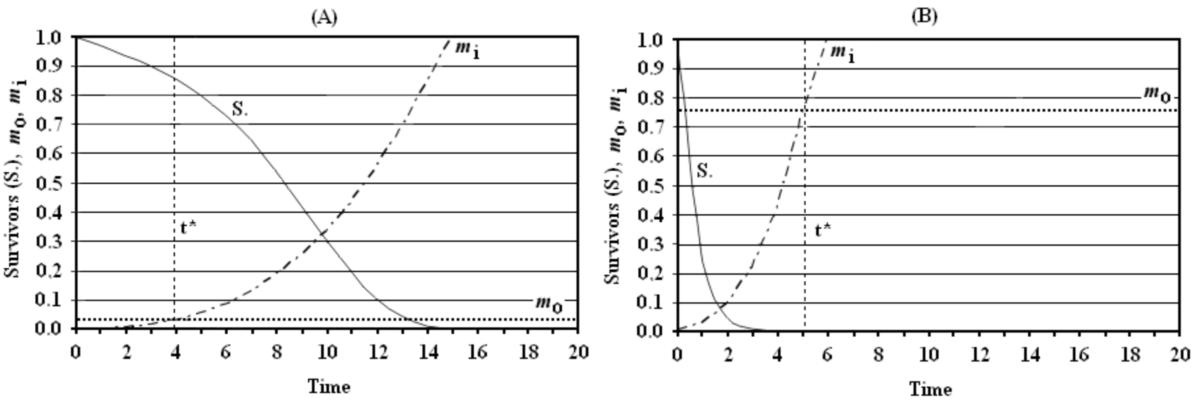

**FIGURE 8**. (A) Life table of *Panthera leo* ($m_0$ = 0.032; α = 0.000252; β = 3). (B) Life table of *Parus major* (M) ($m_0$ = .756; α = .00343; β = 3). In both cases, data are from [2], and figures illustrate life tables and the values of $m_0$ and of $m_i$. The dashed vertical lines indicate the time t* when $m_i$ becomes greater than $m_0$.

Ricklefs plainly states in his discussion (not in the title of his paper!) that this prediction is clearly contradicted by the inverse relation observed between $m_0$ and the proportion of deaths due to $m_i$[2]. As exposed above, the apparent contradictory result of the positive relation between $m_0$ and $m_i$, is explained with a careful analysis of the inverse relation between $\log_{10}(m_0)$, or $m_0$, and the survivors at age t* when the total increment of mortality ($m_i$) becomes greater than $m_0$.

For a theory based on the **adaptive hypothesis**, if there is an optimal value of the ML for certain ecological conditions, this is the result of a balance between the selection for a greater ML and the combined effects of $m_e$ and [c] (see Fig. 3). If we consider two species with a different $m_e$ and the same optimal value of ML, in the species with the greater $m_e$ to have the same value of ML, the factor [c] must have lower efficacy in reducing ML. Therefore, the adaptive hypothesis predicts, as observed, an inverse relation between $m_e$ and [c], alias $m_i$[8,9].

The **historical hypothesis** maintains that the major determinant of life tables is the ancestry, while selective factors have secondary influence. However, data from two subphyla of *cordata* show that life tables are strongly and above all conditioned by selective factors.

## Telomere-Telomerase System

### *Empirical Evidence*

Somatic cell reproduction capacities have well-known limits *in vitro* (Hayflick limit[31,32]) and *in vivo*[33], of which the underlying mechanisms have been defined[34].

Eukaryotic chromosomal DNA is linear and, in the replication, a small portion of one of the two ends (telomeric DNA) is lost[35]. Telomeric DNA, a highly conserved repetitive sequence (TTAGGG in vertebrates, slime molds, trypanosomes, and many other organisms[36]), in general several kilobase pairs long, at each replication shortens[37] by up to a length, disallowing further replications[34].

Enzyme telomerase adds new segments of the repetitive sequence[38] and telomerase introduction in somatic cells "immortalizes" them, i.e., renders them capable of innumerable duplications[39].

There is not a strict relation between the length of telomeric DNA and the number of possible duplications (e.g., mouse telomeric DNA is much longer than human telomeric DNA, but the number of possible duplications is smaller). The length of telomeric DNA is a sort of counter, but its effects are dependent on the relative variation in length and on a species-specific regulation[40].

Telomeric DNA shortening is inversely dependent on the activity of telomerase, which for some cells is always active (cells of the germinal line), for others is always inactive (most human somatic cells), and for others still, is sporadically more or less active in certain conditions[40].

In short, cell duplication capacity is not a simple mechanical outcome of an unsolvable defect in DNA replication and of a telomeric DNA finite length, but one that potentially varies, without an upper limit, from cell to cell, depending on telomere-telomerase regulation; i.e., it is not a phenomenon caused by insurmountable ties, but is a genetically determined and regulated function.

Moreover, with the progressive shortening of telomeric DNA, the expression of many genes, among those usually expressed by the cell, proves to be impaired, altering overall cell functionality and, consequently, the functions of the extracellular matrix and of other near or physiologically interdependent cells. It has been extensively and soundly documented that this decay of cell functions (cell senescence), as well as the progressive reduction of cell duplication capacities (replicative senescence), somehow depends on the relative shortening of telomeric DNA (Fossel's "cell senescence limited model")[40].

Furthermore, the concept of a sharp drop in cell duplication capacities when the shortening of telomeric DNA length exceeds a certain limit has been revised and formulated in a more sophisticated way. The telomere, constituted by the telomeric DNA and a proteinic component, is a dynamic complex with the telomeric DNA oscillating between a capped phase (DNA tied to the proteinic component) and an uncapped phase (DNA not tied). The fraction of time during which telomeric DNA is capped is directly proportional to its relative length. Uncapped telomeric DNA is more vulnerable to the blocking of its duplication capacity. Therefore, replicative senescence is gradual and progressive and not abrupt[41].

Because organism functional efficiency for many species (e.g., vertebrates) depends on a continuous cell turnover, the progressive replicative senescence and the progressive alterations caused by cell senescence bring about a progressive decay of living functions (Fossel's cell senescence general model of aging)[9,40].

This overall decay of functions would certainly be defined by anybody as aging in its evident and extreme manifestations, which we may observe only in artificial conditions of low extrinsic mortality. However, the initial phases of this decay, which can certainly be observed in the wild, mean a limited reduction in fitness; i.e., the fitness decline of an IMICAW species observed in the wild and, therefore, Fossel's cell senescence general model of aging can be reformulated as a cell senescence general model for IMICAW as well.

The **nonadaptive hypothesis** does not predict the existence of mechanisms that are genetically determined and regulated causing fitness decline. The above-mentioned mechanisms can be compatible with the nonadaptive hypothesis, only if an adaptive function is a plausible and exhaustive evolutionary justification for their existence.

A possible purpose for replicative senescence and cell senescence is that of a general defense against the threat of malignant tumors[42,43], in a sort of evolutionary trade-off between aging and cancer restriction[44]. However, this hypothesis does not justify the great differences in duplication limits and in cell overall functionality decay from species to species, unless the risk of malignant tumors is postulated as varying from species to species in direct correlation with the limits imposed to cell duplication capacities and on overall cell functionality by the genetic modulation of the telomere-telomerase system.

About telomerase action and oncogenic risk: old rainbow trout and lobsters, "animals with negligible senescence", have, in the wild, the same levels of telomerase activity as young individuals[45,46] and increasing problems of carcinogenesis at older ages are not plausible for them because, as their definition states, their mortality rates do not increase with age.

Moreover, (1) the decline of duplication capacities and of overall cell functionality weakens immune system efficiency[40], which has, for a long time, been known to be inversely related to cancer incidence[47]; (2) when telomeres are shortened, there is a great vulnerability to cancer because of dysfunctional telomere-induced instability[48,49]; (3) "The role of the telomere in chromosomal stability ... argues that telomerase protects against carcinogenesis ..., especially early in carcinogenesis when genetic stability is critical ..., as well as protecting against aneuploidy and secondary speciation ... . The role of telomerase depends on the stage of malignancy ...; expression is late and permissive, not causal ...”[40] (p. 78, references have been left out from the quotation).

The **adaptive hypothesis** predicts and requires the existence of mechanisms that are genetically determined and regulated, causing the mortality increment, i.e., fitness decline. Therefore, the evidence briefly reported above is not only consistent with adaptive hypothesis, but is also indispensable for its admissibility.

The **historical hypothesis** does not predict the evolution of mechanisms that progressively reduce cell reproduction capacities and overall cell functionality with rates that are largely variable from species to species.

## CONCLUSIONS

Adaptive hypothesis predictions are consistent with empirical evidence. In particular, the adaptive hypothesis predicts the existence of non-IMICAW species, appears indispensable in explaining the observed inverse correlation between extrinsic mortality and the proportion of deaths due to intrinsic mortality, and gives a possible justification for the sophisticated mechanisms limiting and regulating cell duplication capacities and overall cell functionality.

On the contrary, the nonadaptive hypothesis has unlikely explanations for the absence of the IMICAW phenomenon in many species, is strongly contradicted by the observed inverse correlation between extrinsic mortality and the proportion of deaths due to intrinsic mortality, and fails to predict the sophisticated mechanisms that cause replicative senescence and cell senescence and their highly varied regulation.

Moreover, reformulating some well-known theories of aging as theories of IMICAW based on the nonadaptive hypothesis, they suffer from the contradictions of their predictions with observational data (e.g., disposable soma theory predicts a trade-off between reproduction and longevity, but this is not proved by available data for human, primates[50] or any other IMICAW species; the same theory is contradicted by the positive relation of caloric restriction with a greater lifespan[51]; for antagonistic pleiotropic theory no pleiotropic gene is documented in an IMICAW species[2]).

Finally, the historical hypothesis seems likely with the first argument of empirical evidence, but is clearly in difficulty for the other two.

On the whole, only the adaptive hypothesis overcomes the trial of empirical evidence, which has been the only true judge of scientific method since the days of Bacon and Galilei.

This does not mean that whatever theory based on adaptive hypothesis is true because, for such a theory, a precise correlation between its prospected IMICAW-favoring conditions and the presence of the IMICAW phenomenon, and vice versa, its absence in non-IMICAW-favoring conditions, is strictly required.

However, while a theory on the evolutionary meaning of the IMICAW phenomenon based on the adaptive hypothesis has the possibility of being a correct theory, empirical evidence leads us to exclude, or at least to have severe doubts about, theories based on nonadaptive or historical hypotheses.

As a practical consequence of this discussion, if, as empirical data seem to indicate, the progressive decline in fitness is caused by genetically determined and regulated mechanisms, i.e., if the IMICAW phenomenon is a physiologic function, a different modulation of it is a possibility, thus strengthening the position that “the Foreseeable Defeat of Aging Is Not Laughable”[52].
